# Characteristic Cerebrospinal Fluid Cytokine/Chemokine Profiles in Neuromyelitis Optica, Relapsing Remitting or Primary Progressive Multiple Sclerosis

**DOI:** 10.1371/journal.pone.0061835

**Published:** 2013-04-18

**Authors:** Takuya Matsushita, Takahisa Tateishi, Noriko Isobe, Tomomi Yonekawa, Ryo Yamasaki, Dai Matsuse, Hiroyuki Murai, Jun-ichi Kira

**Affiliations:** 1 Department of Neurology, Neurological Institute, Graduate School of Medical Sciences, Kyushu University, Fukuoka, Japan; 2 Department of Neurological Therapeutics, Neurological Institute, Graduate School of Medical Sciences, Kyushu University, Fukuoka, Japan; Research Inst. of Environmental Med., Nagoya Univ., Japan

## Abstract

**Background:**

Differences in cytokine/chemokine profiles among patients with neuromyelitis optica (NMO), relapsing remitting multiple sclerosis (RRMS), and primary progressive MS (PPMS), and the relationships of these profiles with clinical and neuroimaging features are unclear. A greater understanding of these profiles may help in differential diagnosis.

**Methods/Principal Findings:**

We measured 27 cytokines/chemokines and growth factors in CSF collected from 20 patients with NMO, 26 with RRMS, nine with PPMS, and 18 with other non-inflammatory neurological diseases (OND) by multiplexed fluorescent bead-based immunoassay. Interleukin (IL)-17A, IL-6, CXCL8 and CXCL10 levels were significantly higher in NMO patients than in OND and RRMS patients at relapse, while granulocyte-colony stimulating factor (G-CSF) and CCL4 levels were significantly higher in NMO patients than in OND patients. In NMO patients, IL-6 and CXCL8 levels were positively correlated with disability and CSF protein concentration while IL-6, CXCL8, G-CSF, granulocyte-macrophage colony-stimulating factor (GM-CSF) and IFN-γ were positively correlated with CSF neutrophil counts at the time of sample collection. In RRMS patients, IL-6 levels were significantly higher than in OND patients at the relapse phase while CSF cell counts were negatively correlated with the levels of CCL2. Correlation coefficients of cytokines/chemokines in the relapse phase were significantly different in three combinations, IL-6 and GM-CSF, G-CSF and GM-CSF, and GM-CSF and IFN-γ, between RRMS and NMO/NMOSD patients. In PPMS patients, CCL4 and CXCL10 levels were significantly higher than in OND patients.

**Conclusions:**

Our findings suggest distinct cytokine/chemokine alterations in CSF exist among NMO, RRMS and PPMS. In NMO, over-expression of a cluster of Th17- and Th1-related proinflammatory cytokines/chemokines is characteristic, while in PPMS, increased CCL4 and CXCL10 levels may reflect on-going low grade T cell and macrophage/microglia inflammation in the central nervous system. In RRMS, only a mild elevation of proinflammatory cytokines/chemokines was detectable at relapse.

## Introduction

Neuromyelitis optica (NMO) is characterized by severe and selective involvement of the optic nerves and spinal cord, which frequently presents as longitudinally extensive spinal cord lesions (LESCLs) extending over three or more vertebral segments, and neutrophil-predominant pleocytosis of the CSF [1]. The discovery of an NMO-IgG specific for NMO [2] that recognizes the water channel protein aquaporin-4 (AQP4) [3], suggested that the NMO-IgG/anti-AQP4 antibody is the sole pathogenic factor underlying NMO. Pathologically, NMO lesions are characterized by vasculocentric deposition of complement and immunoglobulins, and loss of AQP4 and astrocytes [4], suggesting the involvement of humoral immunity and astrocytic damage in disease etiology. Intraperitoneal injection of immunoglobulin containing anti-AQP4 antibody into T-cell–mediated experimental autoimmune encephalomyelitis (EAE) rats augmented the clinical severity and produced NMO-like lesions [5]. Intracerebral co-injection of anti-AQP4 antibody and human complement produced NMO-like lesions in mice [6]. In an ex vivo study, mouse optic nerves exposed to anti-AQP4 antibody-positive serum showed reduced levels of myelin basic protein [7]. These findings suggested anti-AQP4 antibody induces pathogenic effects. However, anti-AQP-4 antibody injection into young rats with a leaky blood–brain barrier did not induce disease or neuropathological changes in the central nervous system (CNS), despite apparent vascular leakage of human immunoglobulin to the perivascular tissue [8]. Thus, it is still unknown whether NMO is caused by anti-AQP4 antibody and complement alone or whether T cell involvement is required to trigger inflammation. In the present study, we compared cerebrospinal fluid (CSF) cytokine/chemokine profiles among patients with NMO, relapsing remitting multiple sclerosis (RRMS) and primary progressive MS (PPMS), to assess T cell cytokine alterations at relapse, and in remission and progression phases, in distinct demyelinating conditions.

## Methods

### Ethics Statement

This study was approved by the ethics committee of Kyushu University Hospital. Written informed consent was obtained from each participant.

### Participants

All patients were examined in the Department of Neurology at Kyushu University Hospital, Japan, between 2000 and 2008. We determined the presence of anti-AQP4 antibody in sera of all patients using an immunofluorescence method as described previously [9]. CSF samples of 73 patients were available for this study. For diagnosis, we defined NMO spectrum disorder (NMOSD) based on the revised NMO criteria [10] as follows: (1) patients with recurrent optic neuritis or myelitis and fulfilling at least two of the three supportive criteria in revised NMO criteria; and (2) patients with recurrent optic neuritis and myelitis and fulfilling at least one of the three supportive criteria [11]. Thirty-five patients fulfilled McDonald's MS criteria [12] and did not fulfill the revised criteria for NMO or NMOSD. Twenty-six patients had a relapsing-remitting course, and nine patients were diagnosed with PPMS based on McDonald's criteria [12]. Among patients with RRMS, 13 samples were collected at the relapse phase (within one month of the initiation of relapse) and the rest were collected in the remission phase. Fourteen patients fulfilled the revised NMO criteria and six patients were diagnosed with NMOSD based on our definition. One of 20 patients with NMO/NMOSD was negative for anti-AQP4 antibodies while the rest were anti-AQP4 antibody positive. Among them, 16 samples were collected at the relapse phase and the others were collected in the remission phase. In addition, 18 patients with other non-inflammatory neurological diseases (OND) were also enrolled. The OND group included three patients with spinocerebellar degeneration, two each with idiopathic dystonia and psychogenic movement disorders, and one each with alcoholic ataxia, aponeurotic ptosis, corticobasal degeneration, facioscapulohumeral muscular dystrophy, hereditary spastic paraplegia, multiple system atrophy, normal pressure hydrocephalus, reflex sympathetic dystrophy type 2, sleep disturbance, trigeminal neuralgia, and vitamin B12 deficiency. In two patients with NMO/NMOSD, CSF was withdrawn after administration of steroid pulse therapy. Five patients with MS and five with NMO/NMOSD were administered low-to-medium doses of prednisolone (40 mg/day or less), and four patients with MS and one with NMO/NMOSD were administered interferon β-1b (8 million units every other day) at the time of CSF withdrawal. One patient with NMO received monthly high-dose intravenous immunoglobulin therapy. Disability at the time of CSF collection was evaluated according to Kurtzke's Expanded Disability Status Scale (EDSS) scores [13]. The demographic features of the subjects including clinical stages at CSF collection and CSF findings are summarized in Table 1. The female to male ratio was significantly higher in NMO/NMOSD patients than in OND patients (^corr^p = 0.013), while there were no significant differences in age, EDSS scores and disease duration at the time of sample collection among NMO/NMOSD, RRMS and PPMS patients. CSF neutrophil counts at the time of CSF sampling were higher in NMO/NMOSD patients than in MS patients, and maximal longitudinal spinal cord lesion length (vertebrae) was longer in NMO/NMOSD patients than in RRMS and PPMS patients. Oligoclonal IgG band positivity rates were higher in RRMS and PPMS patients than in NMO/NMOSD patients.

**Table 1:** Demographic features of patients.

	NMO/NMOSD (n = 20)	RRMS (n = 26)	PPMS (n = 9)	OND (n = 18)
Female∶Male	17∶3*	17∶9	3∶6	6∶12*
Relapse∶Remission	16∶4	13∶13	NA	NA
Age at sample collection, mean (SD), y	49.1 (11.4)	40.0 (12.8)	39.1 (10.1)	46.3 (17.5)
EDSS at sample collection, mean (SD)	5.6 (2.5)	4.3 (2.1)	4.9 (1.6)	NA
Disease duration at sample collection, mean (SD), y	10.6 (9.7)	8.6 (11.1)	10.8 (9.2)	NA
CSF total protein, mean (SD), mg/dl	46.7 (28.7) (n = 19)	33.2 (18.6)	35.4 (15.6)	NA
CSF cell count, mean (SD), /µl	11.5 (15.3) (n = 19)	3.4 (3.1)	2.8 (2.7)	NA
CSF neutrophil count, mean (SD), /µl	3.2 (6.8)* (n = 19)	0.038 (0.20)*	0.11 (0.33)	NA
IgG index, mean (SD)	0.54 (0.10) (n = 17)	0.69 (0.28) (n = 25)	0.72 (0.22)	NA
Oligoclonal IgG bands	1/18* #	14/25*	8/9#	NA
Longitudinal spinal cord lesion length at time of sample collection, mean (SD), vertebrae	5.8 (5.0)* #	1.7 (2.1)*	1.8 (0.67)#	NA

NA = not applicable; NMO = neuromyelitis optica; NMOSD = neuromyelitis optica spectrum disorder; OND = other non-inflammatory neurological diseases; PPMS = primary progressive multiple sclerosis; RRMS = relapsing remitting multiple sclerosis; CSF = cerebrospinal fluid; SD = standard deviation; EDSS = Expanded Disability Status Scale.

*, # p<0.05.

### Multiplexed fluorescent bead-based immunoassay

CSF samples were obtained from all patients by non-traumatic lumbar puncture, centrifuged within 30 minutes at 800 rpm at 4°C for 5 minutes, and the liquid phase of the CSF was stored at −80°C until use. The levels of 27 cytokines/chemokines and growth factors in the liquid phase of the CSF, namely, interleukin (IL)-1β, IL-2, IL-4, IL-5, IL-6, IL-7, IL-9, IL-10, IL-12 (p70), IL-13, IL-15, IL-17A, interferon (IFN)-γ, tumor necrosis factor (TNF)-α, C-X-C motif ligand (CXCL)8/IL-8, CXCL10/inducible protein-10, C-C motif ligand (CCL)2/macrophage chemoattractant protein-1, CCL3/macrophage inflammatory protein (MIP)-1α, CCL4/MIP-1β, CCL5/RANTES, CCL11/eotaxin, granulocyte colony stimulating factor (G-CSF), granulocyte-macrophage colony stimulating factor (GM-CSF), platelet-derived growth factor (PDGF) bb, basic fibroblast growth factor (bFGF), vascular endothelial growth factor (VEGF), and IL-1 receptor antagonist (IL-1ra), were measured as described previously [14]. The Bio-Plex Cytokine Assay System (Bio-Rad Laboratories, Hercules, CA) was used according to the manufacturer's instructions. Cytokine concentrations were calculated by reference to a standard curve for each molecule derived using various concentrations of the standards assayed in the same manner as the CSF samples. The detection limit for each molecule was determined by the recovery of the corresponding standard, and the lowest values with more than 70% recovery were set as the lower detection limits. All samples were analyzed in duplicate. For the IL-17 assay, all samples were diluted eight-fold with artificial CSF (124 mM NaCl, 3 mM KCl, 1.25 mM KH_2_PO_4_, 10 mM glucose, 2 mM CaCl_2_, 26 mM NaHCO_3_, 1 mM MgSO_4_, 2% BSA) to minimize the influence of interfering proteins. All other samples were measured undiluted.

### Statistical analysis

Cytokines/chemokines whose detection rates were less than 10 percent were excluded from the analyses. Statistical analyses of cytokine levels were performed using the Kruskal–Wallis test and the p values were corrected by Benjamini-Hochberg method. For cytokines/chemokines that had less than 0.05 of corrected *p* values in the Kruskal-Wallis test, the Steel–Dwass test was used to determine the significance of differences between pairs of groups. The Spearman rank correlation coefficient was used for statistical analyses of correlations between cytokines, and between cytokine levels and clinical parameters in the demyelinating disease groups. The *p* values were corrected by the Benjamini-Hochberg method. To compare two Spearman correlation coefficients, the coefficients were transformed with Fisher Z-transformation and the difference was used to determine the level of significance. The threshold for significance was set at *p*<0.05. All calculations were performed by R.

## Results

### Comparison of CSF cytokine/chemokine levels among NMO, RRMS, PPMS and OND patients at relapse or remission phase

**Figure 1:**
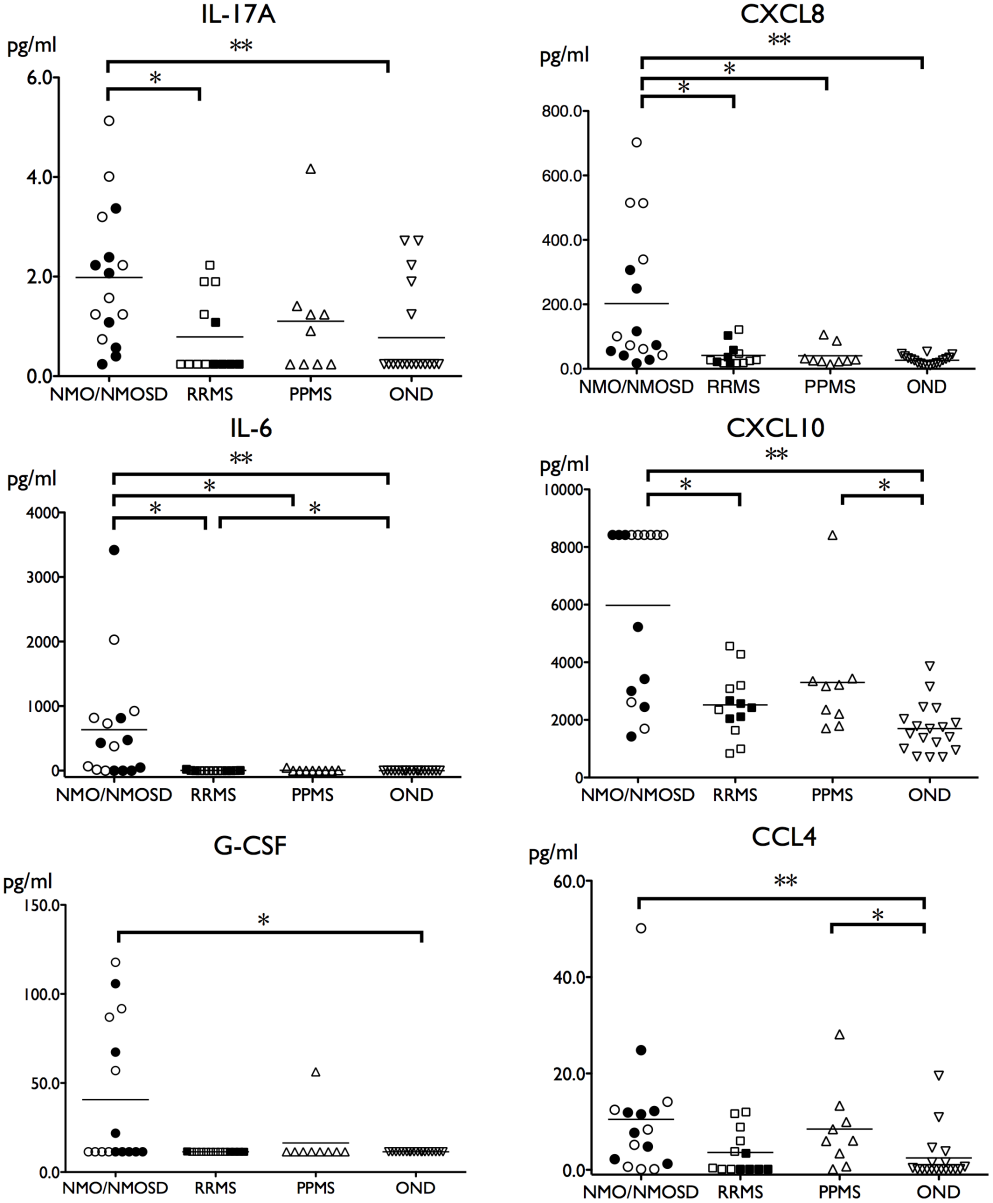
Cytokine and chemokine levels in CSF from patients with NMO/NMOSD, RRMS, PPMS and OND. In NMO/NMOSD patients, the levels of IL-17A, CXCL8, IL-6, CXCL10, G-CSF and CCL4 were higher than in the relapse phase. Closed circles and rectangles in NMO/NMOSD and RRMS groups indicate patients were receiving immunotherapy (corticosteroids, interferon-β, or high-dose intravenous immunoglobulin) at the time of CSF collection. Cytokines that did not show any significant changes are not shown. The lower detection limits were as follows: 0.24 pg/mL for IL-17A, 2.9 pg/mL for CXCL8, 0.24 pg/mL for IL-6, 10.1 pg/mL for CXCL10, 11.4 pg/mL for G-CSF and 0.14 pg/mL for CCL4. The upper detection limit for CXCL10 was 8420 pg/mL. *p<0.05, **p<0.01. The number of subjects per group was 16 in NMO/NMOSD, 13 in RRMS, 9 in PPMS, and 18 in OND. NMO = neuromyelitis optica; NMOSD = neuromyelitis optica spectrum disorder; OND = other non-inflammatory neurological diseases; PPMS = primary progressive multiple sclerosis, RRMS = relapsing remitting multiple sclerosis.

Because the detection rates of IL-1β, IL-2, IL-4, IL-5, IL-7, IL-10, IL-12, IL-13, TNF-α, bFGF, CCL3 and PDGF were <10% in all groups, we excluded these cytokines/chemokines from further analyses. At relapse, statistically significant differences by Kruskal–Wallis test were noted in the levels of IL-6 (*^corr^p* = 0.00014), CXCL8 (0.0017), IL-17A (0.010), G-CSF (0.010), CCL4 (0.010), and CXCL10 (0.00031) among patients with NMO, RRMS, PPMS and OND after correction for multiple tests by the Benjamini-Hochberg method (Fig. 1). No cytokine/chemokine levels were significantly different among these groups in the remission phase. Among those cytokines that showed significant differences by Kruskal–Wallis test in the relapse phase, IL-17A, IL-6, CXCL8, G-CSF, CCL4, and CXCL10 levels were higher in NMO/NMOSD patients than in OND patients assessed by the Steel-Dwass test (IL-17A: p = 0.0074; IL-6: p = 0.000076; CXCL8: *p* = 0.0003; G-CSF: p = 0.011; CCL4: p = 0.0092; CXCL10: p = 0.00022). IL-17A, IL-6, CXCL8 and CXCL10 levels were also higher in NMO/NMOSD patients than in RRMS patients (IL-17A: p = 0.024; IL-6: p = 0.012; CXCL8: p = 0.019; CXCL10: p = 0.019). IL-6 and CXCL8 levels were higher in NMO/NMOSD patients than in PPMS patients (IL-6: p = 0.020; CXCL8: p = 0.039). IL-6 levels were higher in RRMS patients than in OND patients (p = 0.025). CCL4 and CXCL10 levels were higher in PPMS patients than in OND patients (CCL4: *p* = 0.045; CXCL10: *p* = 0.024). Comparison of levels of 15 cytokines/chemokines analyzed between NMO and NMOSD patients did not show any statistically significant differences. Excluding cases who had received therapy, levels of IL-6 and CXCL8 were higher in NMO/NMOSD patients than in MS, PPMS and OND patients (IL-6: *p* = 0.016, 0.014, and 0.000044; CXCL8: *p* = 0.024, 0.027, and 0.0007, respectively), and levels of IL-17A and G-CSF were significantly higher in NMO/NMOSD patients compared with OND patients at relapse (*p* = 0.012 and 0.0079, respectively).

### Comparison of cytokine/chemokine levels between relapse and remission phases in NMO/NMOSD and RRMS patients

Among the cytokines/chemokines examined, only IL-6 and CXCL8 levels were higher in the relapse phase than in the remission phase in patients with NMO/NMOSD, but the differences were not statistically significant after multiple test corrections were made (*^uncorr^p* = 0.022 and 0.012, respectively). In patients with RRMS, CCL2, IL-9 and IL-15 levels were higher in the remission phase than in the relapse phase (*^uncorr^p* = 0.0077, 0.031, and 0.0048, respectively), but again the differences were not statistically significant after multiple test correction (Fig. S1). CCL11 levels also had a tendency to be higher in the remission phase than in the relapse phase (*^uncorr^p* = 0.0578).

### Relationship between elevated cytokine/chemokine levels and clinical parameters in NMO/NMOSD and RRMS patients

We then analyzed potential correlations between elevated cytokine/chemokine levels and clinical parameters including EDSS score, CSF protein concentration, CSF cell count, CSF neutrophil counts, IgG index, and maximal spinal cord lesion length at the time of sample collection. Among all cytokine/chemokine and clinical parameters analyzed in the relapse phase, IL-6 and CXCL8 were positively correlated with EDSS score (IL-6: r = 0.72, *^corr^p* = 0.012; CXCL8: r = 0.81, *^corr^p* = 0.0020) (Table 2). With regard to imaging, IL-6 and G-CSF levels were positively correlated with maximal spinal cord lesion length at the time of sample collection (IL-6: r = 0.47, *^uncorr^p* = 0.035; G-CSF: r = 0.47, *^uncorr^p* = 0.038), but the correlation was not significant after correction for multiple tests. CSF protein concentration was positively correlated with IL-6 and CXCL8 levels (IL-6: r = 0.69, *^corr^p* = 0.024; CXCL8: r = 0.76, *^corr^p* = 0.011). CSF cell counts were positively correlated with IL-6 and G-CSF levels (IL-6: r = 0.75, *^corr^p* = 0.012; G-CSF: r = 0.70, *^corr^p* = 0.019). CSF neutrophil counts were positively correlated with the levels of IL-6, CXCL8, G-CSF, GM-CSF and IFN-γ (IL-6: r = 0.81, *^corr^p* = 9.6E-4; CXCL8: r = 0.61, *^corr^p* = 0.043; G-CSF: r = 0.89, *^corr^p* = 7.5E-5; GM-CSF: r = 0.64, *^corr^p* = 0.041; IFN-γ: r = 0.60, *^corr^p* = 0.043) (Table 2). Among patients with RRMS in the relapse phase, CSF cell counts were negatively correlated with CCL2 levels (r = −0.75, *^corr^p* = 0.047). In the remission phase, no significant correlation between clinical parameters and cytokines/chemokines in RRMS or NMO/NMOSD was observed.

**Table 2:** Relationship of cytokine and chemokine levels with clinical parameters in patients with RRMS and NMO/NMOSD in the relapse phase.

RRMS in the relapse phase			
Clinical parameter	Cytokine/chemokine	Rho	*^corr^P*
CSF cell count (/µl)	CCL2	−0.75	0.047

NMO/NMOSD in the relapse phase			
Clinical parameter	Cytokine/chemokine	rho	*^corr^P*
EDSS score	IL-6	0.72	0.012
	CXCL8	0.81	0.002
CSF protein concentration	IL-6	0.69	0.024
	CXCL8	0.76	0.011
CSF cell count (/µl)	IL-6	0.75	0.012
	G-CSF	0.70	0.019
CSF neutrophil count (/µl)	IL-6	0.81	9.6E-04
	CXCL8	0.61	0.043
	G-CSF	0.89	7.5E-05
	GM-CSF	0.64	0.041
	IFN-γ	0.60	0.043

EDSS = Expanded Disability Status Scale; NMO = neuromyelitis optica; NMOSD = neuromyelitis optica spectrum disorder; RRMS = relapsing remitting multiple sclerosis; CSF, cerebrospinal fluid. rho = Spearman's correlation coefficient, *^corr^p* = corrected p value by Benjamini-Hochberg method.

At the time of CSF collection during the relapse phase of NMO/NMOSD, seven patients were receiving corticosteroids and one interferon beta (IFN-β) while eight had no immunotherapy. Comparison of untreated NMO/NMOSD patients or those receiving corticosteroids (excluding one patient receiving IFN-β therapy) demonstrated no statistically significant differences in all cytokine/chemokine levels tested (*^uncorr^p* = 0.072–0.87). In contrast, during the relapse phase of RRMS, IL-9 and IL-15 levels were higher in four patients treated with prednisolone than in eight patients without immunotherapy (*^uncorr^p* = 0.028 and 0.0081, respectively). In the remission phase, the sample size was too small to compare between patients with and without immunotherapy or those receiving other disease-modifying therapies (DMTs).

### Correlation between cytokines/chemokines in patients with NMO/NMOSD or RRMS according to the clinical phase

**Figure 2:**
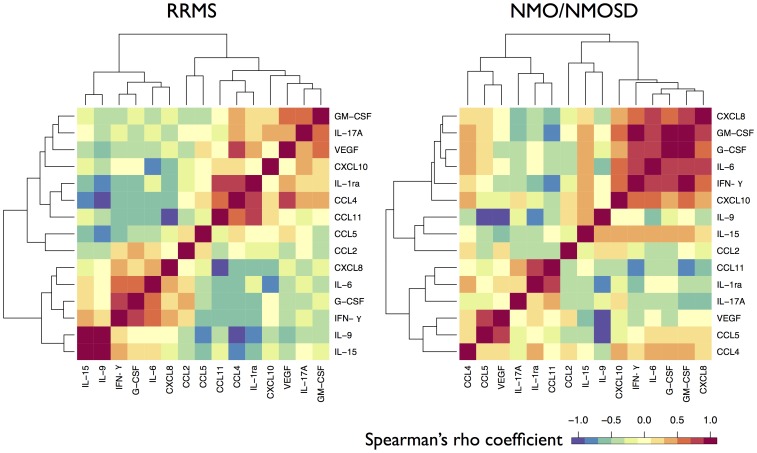
Correlation of cytokine and chemokine levels in patients with RRMS and NMO/NMOSD in the relapse phase. Among the cytokines and chemokines analyzed, distances of each pair of cytokines/chemokines, based on Spearman's correlation coefficient, of RRMS and NMO/NMOSD in the relapse phase were shown as a heatmap. NMO = neuromyelitis optica; NMOSD = neuromyelitis optica spectrum disorder; RRMS = relapsing remitting multiple sclerosis.

Among 15 cytokines/chemokines analyzed, correlations of all combinations were tested in patients with RRMS or NMO/NMOSD in the relapse phase. Three combinations in RRMS and 13 combinations in NMO/NMOSD had significant correlations after multiple test (105) correction (Table 3, Fig. 2). In OND, five pairs of molecules had significant correlations. Rho correlation coefficients of cytokines/chemokines between RRMS and NMO/NMOSD in the relapse phase were significantly different in three combinations (IL-6 and GM-CSF: *^corr^p* = 0.034; G-CSF and GM-CSF: *^corr^p* = 0.034; GM-CSF and IFN-γ: *^corr^p* = 0.034). In RRMS patients, two pairs had significant correlations in the remission phase. There was no significant difference of correlation coefficient in pairs between relapse and remission phases of RRMS.

**Table 3:** Correlations of cytokines/chemokines in RRMS and NMO/NMOSD in the relapse phase.

	Status	Cytokines/Chemokines combination	rho	*^corr^P*
RRMS	Relapse	CCL4 - IL-1ra	0.82	2.8E-02
		IL-1ra - CCL11	0.81	4.3E-02
		IL-9 - IL-15	0.87	1.3E-02
	Remission	IL-9 - IL-15	0.96	0
		IL-9 - CCL5	−0.81	2.9E-02
NMO/NMOSD	Relapse	IL-6 - CXCL8	0.82	1.6E-03
		IL-6 - G-CSF	0.85	1.1E-03
		IL-6 - GM-CSF	0.82	1.6E-03
		IL-6 - IFN-γ	0.75	9.9E-03
		IL-6 - CXCL10	0.68	3.2E-02
		CXCL8 - G-CSF	0.71	2.0E-02
		CXCL8 - GM-CSF	0.76	9.9E-03
		CXCL8 - IFN-γ	0.68	3.1E-02
		G-CSF - GM-CSF	0.86	8.3E-04
		G-CSF - IFN-γ	0.83	1.6E-03
		GM-CSF - IFN-γ	0.87	8.3E-04
		IL-1ra - CCL11	0.75	9.9E-03
		CCL5 - VEGF	0.73	1.5E-02
OND		CCL2 - CXCL10	0.69	4.1E-02
		IL-1ra - CCL11	0.66	4.1E-02
		IL-1ra - VEGF	0.72	2.6E-02
		IL-9 - IL-15	0.91	0
		CCL11 - VEGF	0.71	2.6E-02

NMO = neuromyelitis optica; NMOSD = neuromyelitis optica spectrum disorder; OND = other non-inflammatory neurological diseases; RRMS = relapsing remitting multiple sclerosis. rho = Spearman's correlation coefficient, *^corr^p* = corrected p value by Benjamini-Hochberg method.

## Discussion

We previously reported high levels of IL-17 and CXCL8 in the CSF of Japanese patients with the opticospinal form of MS [14], [15]. In this study, applying established NMO/NMOSD criteria for idiopathic CNS demyelinating diseases, including anti-AQP4 antibody positivity, we observed higher levels of IL-17A, IL-6, CXCL8, G-CSF, CCL4 and CXCL10 in the CSF of patients with NMO/NMOSD in the relapse phase than in the CSF of OND patients. Furthermore, CSF levels of IL-17A, IL-6, CXCL8, and CXCL10 were higher in NMO/NMOSD patients than in patients with RRMS. Elevation of pro-inflammatory cytokines/chemokines, such as IL-6, IL-1β, CXCL8, G-CSF, CCL4, and CXCL10, were previously reported in NMO patients [16], [17], [18]. Our study shows for the first time an elevation of IL-17A in the CSF of NMO patients and the correlation of elevated downstream pro-inflammatory cytokines/chemokines with various clinical and laboratory parameters of disease activity. When the increase of proinflammatory cytokines such as IL-6 and CXCL8 during the relapse phase was compared with the remission phase in NMO/NMOSD patients, statistical significance between the groups was not observed after correction for multiple comparisons, probably due to the small sample size used. Because of difficulty obtaining remission phase CSF samples, future studies should strictly compare cytokine/chemokine levels between relapse and remission phases in NMO/NMOSD using increased sample numbers.

IL-17 is thought to be a key factor for autoimmune diseases based on EAE studies [19], [20] and collagen-induced arthritis [21], [22], a model of rheumatoid arthritis (RA). IL-17 promotes the production of IL-6, CXCL8, and G-CSF that augments inflammation by endothelial cells, fibroblasts, macrophages and astrocytes. Synovial fluid from RA patients had elevated levels of IL-6 [23], CXCL8 [24], and IL-17 [25], [26], reflecting inflammatory amplification. NMO is often a complication of collagen diseases [9], [27]. The similarity of the cytokine/chemokine milieu in the CSF of NMO patients and synovial fluid of RA patients indicates a similar inflammatory cascade occurs in both conditions, suggesting IL-17 may play a critical role in disease pathogenesis. The source of IL-17 in the CSF of NMO patients is not clear. However, as other T cell cytokines were increased, these findings suggest the involvement of T cells, especially IL-17-producing helper T cells (Th17), in the induction of relapse in NMO, in addition to antibody-mediated tissue destruction. IFN-β ameliorated EAE symptoms induced by Th1 cells but exacerbated EAE disease induced by Th17 cells [28]. EAE induced by Th17 cells promotes neutrophil infiltration into the optic nerve and spinal cord, unlike EAE induced by Th1 cells. Blockade of neutrophil elastase ameliorated Th17-induced EAE [29]. IFN-β therapy is generally ineffective in NMO and can exacerbate disease in some cases [30], [31]. Because NMO lesions frequently have T cell-predominant perivascular cuffing [32], Th17 cells may have an important role in augmenting inflammation in NMO, a process that is not ameliorated by IFN-β. Recently, administration with monoclonal anti-IL-17A antibodies demonstrated efficacy for psoriasis [33], [34], where IL-17A is thought to play a critical role [35]. Accordingly, IL-17A blocking therapy by neutralizing antibodies may also be expected to be beneficial in NMO.

The degree of disability of patients was positively correlated with the concentrations of cytokines/chemokines downstream of IL-17, namely, IL-6 and CXCL8, a potent chemoattractant for neutrophils. We observed strong correlations between CSF neutrophil counts and the levels of these cytokines/chemokines. Neutrophil infiltration of active lesions is a characteristic pathological feature in NMO [36]. Thus, patient disability and the severity of spinal cord damage may be related to the infiltration of neutrophils recruited by CXCL8, G-CSF and GM-CSF.

The CSF of RRMS patients showed only a mild elevation of pro-inflammatory cytokines/chemokines, such as IL-6, in the relapse phase, which was not as prominent as that in NMO patients. In contrast, CCL2, IL-9 and IL-15 levels were greater in the remission phase than in the relapse phase. In addition, we showed for the first time to the best of our knowledge, a significant negative correlation between CCL2 levels and CSF cell counts in the relapse phase of RRMS. These cytokines/chemokines might be involved in down-regulating inflammation and sustaining remission in RRMS patients. Decreased CSF CCL2 levels in the active phase of RRMS and during recovery after methylprednisolone therapy were previously reported [37]. CCL2 primes T cells towards a type 2 helper T (Th2) phenotype, while IL-9 and CCL11 belong to the Th2 family of cytokines/chemokines. Accordingly, the current study suggests that Th2 activity may contribute to sustaining the remission state in RRMS patients. During the relapse phase, IL-9 and IL-15 levels were higher in RRMS patients receiving prednisolone treatment than those without immunotherapy, similar to the elevated levels of IL-9 and IL-15 in the remission phase. Therefore, treatment with corticosteroids may convert the acute phase CSF cytokine/chemokine profile towards the remission phase profile in RRMS. Further future studies are required to confirm this issue using a large number of relapse and remission phase CSF samples.

Correlation of pairs of cytokines/chemokines showed different patterns between NMO/NMOSD and RRMS. In NMO/NMOSD, correlations among proinflammatory cytokines/chemokines, (CXCL8, GM-CSF, G-CSF, IL-6, IFN-γ and CXCL10) were prominent in the relapse phase, while in RRMS, correlations among Th2 and potential anti-inflammatory cytokines/chemokines, (IL-1ra, CCL4, CCL11, IL-9, and IL-15) were observed in the relapse and/or remission phases. The latter may suggest that the anti-inflammatory cytokine network is still operative in RRMS patients possibly as a host defense mechanism. Augmentation of a cluster of Th17/Th1 proinflammatory cytokines/chemokines including CXCL8, G-CSF and GMCSF appeared to be characteristic of NMO/NMOSD inflammation, which may contribute to the mobilization of neutrophils into the CNS and the subsequent tissue destruction in NMO [36]. In contrast, alterations in levels of anti-inflammatory cytokine/chemokine clusters in the CSF of RRMS patients were observed. IL-15 is an essential cytokine that maintains CD8-positive memory T cells. Elevation of IL-15 in MS CSF was previously reported, but its role in MS is still unclear. It was previously thought to contribute to tissue damage by activating CD8-positive T cells [38], [39]. However, IL-15 also attenuates the cytotoxicity of CD8-positive T cells via enhancement of the killer-inhibitory receptor CD94/NKG2A [40]. Elevation of IL-15 together with other Th2 and anti-inflammatory cytokines/chemokines may suggest its potential protective function in RRMS.

Few reports have demonstrated cytokine/chemokine increases in the CSF of PPMS patients such as CXCL13, a B cell chemoattractant [41], IL-10, an anti-inflammatory cytokine [42], and TNF-α [42]. To the best of our knowledge, we have shown for the first time that CCL4 and CXCL10 levels were significantly higher in PPMS patients than in OND patients. CXCL10 is a downstream cytokine of IFN-γ secreted by Th1 cells. CCL4 is a macrophage chemoattractant and -activating cytokine produced by macrophage/microglia [43]. In MS lesions, enhanced expression of CCL4 and infiltration of T cells bearing CCL4 receptor, CCR5, were observed [44], [45]. In PPMS, a mild global inflammation consisting of microglial activation and diffuse low-level T cell activation become prominent [46]. Thus, increased CCL4 and CXCL10 levels in PPMS might reflect an on-going inflammatory process induced by T cells and macrophage/microglia compartmentalized in the CNS.

## Supporting Information

Figure S1 **Changes in CSF cytokine and chemokine levels between relapse and remission phases in NMO/NMOSD and RRMS patients.** Bars indicate the mean concentration of each group. Closed circles and rectangles indicate patients receiving immunotherapy (corticosteroids, interferon-β, or high-dose intravenous immunoglobulin) at the time of CSF collection. Cytokines that did not show significant changes before correction for multiple tests are not shown. The lower detection limits were as follows: 0.65 pg/mL for CCL2, 0.72 pg/mL for IL-9, and 1.09 pg/mL for IL-15. *^uncorr^p<0.05, **^uncorr^p<0.01. NMO = neuromyelitis optica; NMOSD = neuromyelitis optica spectrum disorder; RRMS = relapsing remitting multiple sclerosis; CSF = cerebrospinal fluid.
(TIFF)
