# Supplementary figures and images for: Characteristic Cerebrospinal Fluid Cytokine/Chemokine Profiles in Neuromyelitis Optica, Relapsing Remitting or Primary Progressive Multiple Sclerosis
Source: PLoS One. 2013 Apr 18;8(4):e61835. doi: 10.1371/journal.pone.0061835 (PMC3630114; doi:10.1371/journal.pone.0061835)

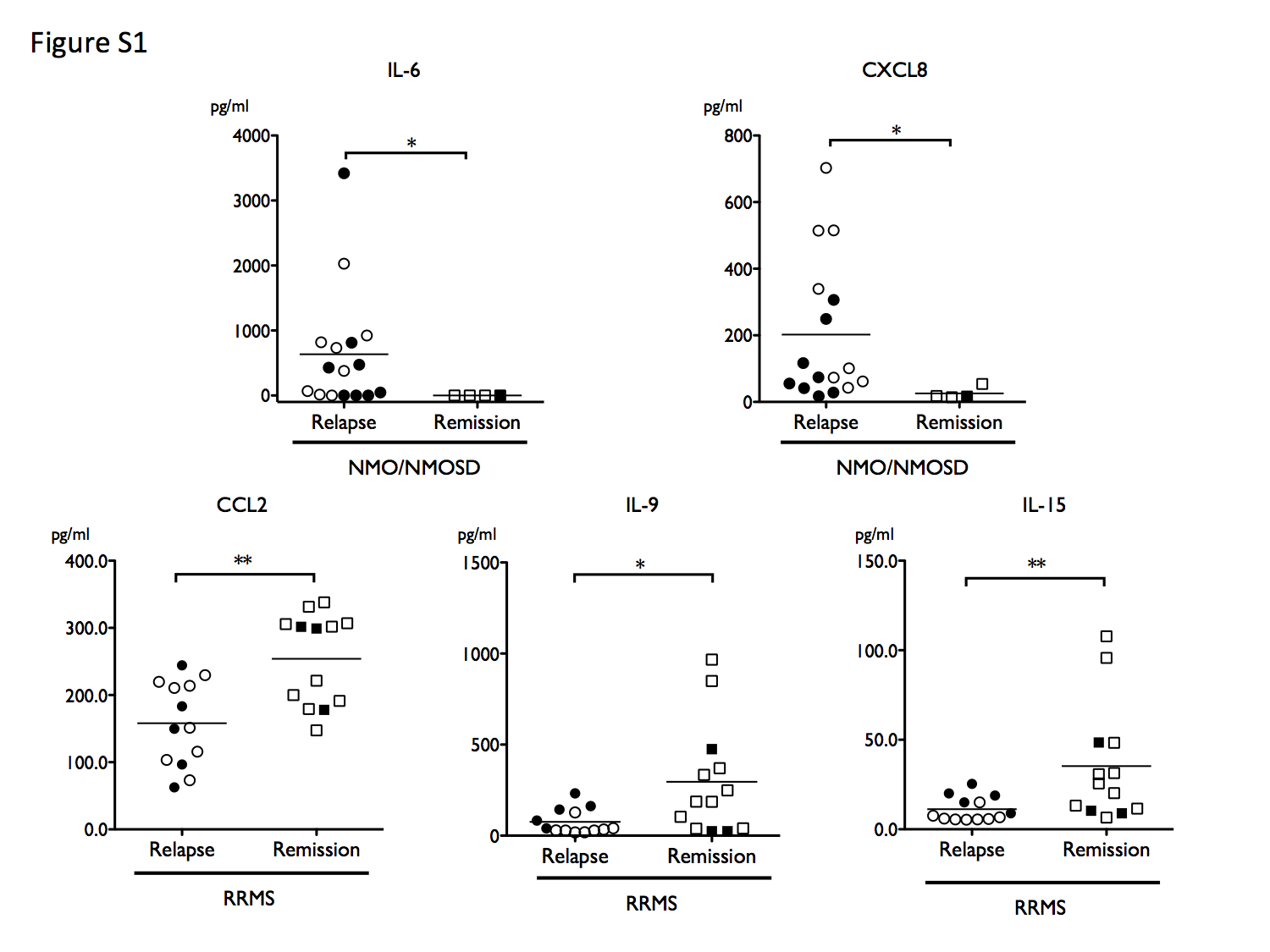

Supplement: Figure S1 — Changes in CSF cytokine and chemokine levels between relapse and remission phases in NMO/NMOSD and RRMS patients. Bars indicate the mean concentration of each group. Closed circles and rectangles indicate patients receiving immunotherapy (corticosteroids, interferon-β, or high-dose intravenous immunoglobulin) at the time of CSF collection. Cytokines that did not show significant changes before correction for multiple tests are not shown. The lower detection limits were as follows: 0.65 pg/mL for CCL2, 0.72 pg/mL for IL-9, and 1.09 pg/mL for IL-15. *uncorrp<0.05, **uncorrp<0.01. NMO = neuromyelitis optica; NMOSD = neuromyelitis optica spectrum disorder; RRMS = relapsing remitting multiple sclerosis; CSF = cerebrospinal fluid. (TIFF) [file pone.0061835.s001.tif]
